# Bigotry and Oppressive Laws in Africa Drive HIV in Men Who Have Sex with Men

**DOI:** 10.1371/journal.pmed.1001471

**Published:** 2013-06-18

**Authors:** Jerome Amir Singh

**Affiliations:** 1 Centre for the AIDS Programme of Research in South Africa (CAPRISA), University of Kwazulu-Natal, Durban, South Africa; 2 Dalla Lana School of Public Health, Joint Centre for Bioethics, and Sandra Rotman Centre, University of Toronto, Toronto, Canada

## Abstract

Jerome Singh discusses the findings of a new study by Rachel Jewkes and colleagues in the context of cultural and legal barriers hindering access for African men who have sex with men to HIV-related health services.

*Please see later in the article for the Editors' Summary*

**Linked Research Article**

This Perspective discusses the following new study published in *PLOS Medicine*:

Dunkle KL, Jewkes RK, Murdock DW, Sikweyiya Y, Morrell R (2013) Prevalence of Consensual Male–Male Sex and Sexual Violence, and Associations with HIV in South Africa: A Population-Based Cross-Sectional Study. PLoS Med 10(6): e1001472. doi:10.1371/journal.pmed.1001472

Using a method that offered complete privacy to participants, Rachel Jewkes and colleagues conducted a survey among South African men about their lifetime same-sex experiences.

The study in this week's issue of *PLOS Medicine* by Jewkes and colleagues on the prevalence of consensual male-male sexual activity and non-consensual male-on-male sexual violence, and their respective associations with HIV infection in South Africa [1], makes an important contribution to the dearth of literature on population-based HIV prevalence amongst men who have sex with men (MSM) in the African context. The paper highlights several important findings, including that HIV prevalence amongst South African MSM also has public health implications for South African women, given high levels of bisexuality and sexual concurrency amongst South African MSM. Assuming these findings are generalizable to the rest of sub-Saharan Africa, addressing the health needs of African MSM will require policymakers to meaningfully address significant socio-cultural and legal barriers that hinder access by MSM to HIV-related health services. Failing to do so will fuel the spread of HIV in African men who engage in consensual male-male sexual activity and/or who are victims of male-on-male sexual violence.

## Socio-Cultural Barriers

Open bigotry against homosexuals by such African leaders as Kenya's former President Daniel arap Moi, Uganda's current President Yoweri Museveni, and Zimbabwe's current President Robert Mugabe have hardened views by some African traditionalists that homosexuality is “un-African" [2],[3]. Such attitudes have stigmatised and spurred violence against African MSM and have deterred their access to health facilities [4]–[6]. African traditionalists, politicians, and religious leaders must appreciate that inciting violence and perpetuating African MSM denialism and bigotry breeds stigma and ostracism against MSM, which drives consensual MSM activities underground. Such behaviour also condemns male-on-male sexual assault victims to shameful silence. The end result is that men who engage in consensual sexual activities, and men who are sexually assaulted, are denied the opportunity to access vital HIV-related health services. Such missed opportunities to manage HIV and other sexually transmitted infections in these vulnerable populations also hold major public health implications for female sexual partners of bisexual MSM and male-on-male sexual assault survivors.

## Legal Barriers

Some African countries, such as South Africa, Cape Verde, the Central African Republic, Gabon, Guinea-Bissau, Malawi, Mauritius, Rwanda, São Tomé and Principe, and Swaziland, oppose criminalising homosexuality, and have decriminalised, or pledged to decriminalise, consensual same-sex sexual activities [7]. In many others, however, such acts are criminal offences punishable by fine, imprisonment (including life imprisonment in the case of Tanzania), and the death penalty (i.e., in the Sudan, Mauritania, and Northern Nigeria) [3]. Disturbingly and ironically, such laws may be the products of post-colonial states whose citizens experienced systemic human rights abuses during colonialism, and these countries have since gone on to ratify the Universal Declaration of Human Rights [8], the International Covenant on Civil and Political Rights [9], and the African Charter on Human and People's Rights [10], all of which prohibit unfair discrimination.

In some African countries, certain statutes in combination create an explosive cocktail of discrimination against MSM and serve to discourage MSM from accessing HIV-related health services. In Kenya, for example, the country's HIV and AIDS Prevention and Control Act of 2006 imposes a positive obligation on those who become aware of their HIV-infected status to disclose such status to their sexual partners. This disclosure obligation could potentially deter the uptake of HIV counselling and testing services, and well as nascent HIV self-testing technologies, amongst MSM communities because HIV-positive men would not only have to disclose their HIV status to their female (and male) sexual partners, but in the case of their female sexual partners, they also could inadvertently expose the closeted MSM's same-sex sexual activities. This disclosure would be detrimental to Kenyan MSM communities given that in Kenya's Penal code, same-sex sexual activities constitute an offence punishable by up to 14 years of imprisonment. Kenyan MSM diagnosed with HIV are thus faced with the choice of engaging in deliberate non-disclosure or lies of their HIV status and of their sexuality, to avoid the associated criminal implications of either disclosure. However, non-disclosure of HIV status to sexual partners can be punishable by a fine and/or imprisonment of up to 7 years, in accordance with the country's HIV and AIDS Prevention and Control Act of 2006. This legislation also permits health personnel to make involuntary HIV-status disclosures to sexual partners of the index patient. Such laws effectively deter MSM communities from accessing HIV counseling and testing, prevention, treatment, and care programmes, and merit reconsideration on public health grounds.

Jewkes and colleagues' findings on the prevalence of male-on-male sexual violence are also significant as they highlight shortcomings in the criminal justice systems of many African countries [1]. While the study includes reference to “male-on-male rape", no such crime exists in many African countries. For example, the common law crime of “rape" in South Africa only governs unlawful and intentional vaginal sex with a woman without consent, thus precluding non-consensual penetrative sexual acts between men. Historically, as a result of this narrow definition, following the post-apartheid repeal of sodomy laws, male-on-male perpetrators of penetrative sexual assault could not be charged with the common law crime of “rape" in South Africa. Instead, such perpetrators were charged with the lesser offence of “indecent assault", which carries a lighter penalty than rape. As a result of this loophole, South Africa's legislature moved in 2007 to introduce the statutory offences of “sexual penetration" and “sexual violation", which cover a wide range of non-consensual sexual acts, including male-on-male anal and oral sex [11]. Post-exposure prophylaxis (PEP) is offered to sexual assault survivors in South Africa [12], regardless of sex or sexual orientation. Other African countries should consider decriminalising homosexuality, reforming their legal systems to cater for all forms of non-consensual sexual acts, and providing PEP to male (and female) survivors of sexual assault, on human rights and ethical grounds. Authorities should also consider providing safe sex counselling, condoms, HIV counseling and testing, and HIV treatment to infected male prisoners, as well as PEP and sensitive mental health support to sexual assault survivors in detention facilities.

## Conclusion

The study by Jewkes and colleagues highlights the need for further MSM-related HIV research in African contexts, particularly at the population level. Their findings also emphasise the need for authorities to cater to the health needs of male-on-male sexual violence survivors, including those in detention facilities, as well as to facilitate their access to the health system and HIV-related health services. High bisexuality and sexual concurrency amongst African MSM also highlights the necessity of considering the health needs of MSM communities and, where relevant, their female sexual partners. To this end, African authorities have a duty to actively address the socio-cultural and legal barriers that stigmatize and hinder MSM access to HIV-related health services. Doing so should be regarded as a human rights and ethics imperative, regardless of prevailing social, cultural, religious, policy, and legal norms.

## Abbreviations

MSM: men who have sex with men
PEP: post-exposure prophylaxis
